# Ultrasound remission can predict future good structural outcome in collagen-induced arthritis rats

**DOI:** 10.1038/s41598-019-49948-7

**Published:** 2019-09-16

**Authors:** Wenxue Li, Yiqun Liu, Jiaan Zhu, Arong Bilig, Fang Liu, Zheng Chen

**Affiliations:** Department of Ultrasound, Peking University People’ s Hospital, Beijing, China

**Keywords:** Rheumatoid arthritis, Risk factors

## Abstract

Regarding the persistence of subclinical synovitis, the concept of ultrasound remission has been proposed in addition to clinical remission. The present study aims to explore whether ultrasound remission has predictive value and ultrasound remission at which time point has predictive value for good structural outcome. Collagen-induced arthritis (CIA) was induced in 32 rats by immunizing with bovine type II collagen. Twenty-four CIA rats were treated with rhTNFR:Fc, and 8 rats were left untreated. Ultrasonography was performed to assess synovial hypertrophy, power Doppler (PD) signal, and bone erosion of the ankle joints of both hindpaws every week following the booster immunization. In the treated group, the scores for synovial hypertrophy, PD signal and bone erosions decreased from baseline to the end. Synovial hypertrophy, PD signal, and bone erosion at baseline were not significantly associated with good structural outcome. Ultrasound remission from 4 to 6 weeks after treatment was significantly associated with good outcome and had the highest area under the curve, sensitivity, specificity, and positive and negative predictive values. Therefore, we conclude that ultrasound remission from 4 to 6 weeks after treatment has a high value for predicting good structural outcome in CIA rats.

## Introduction

Rheumatoid arthritis (RA) is the most common inflammatory joint disorder that causes progressive joint damage and functional disability^1^. The primary goal of treating patients with RA is to maximize long-term health-related quality of life by reaching the therapeutic target of remission or low-disease activity^2^. Although the current clinical remission criteria are associated with less radiographic joint damage^3^, progressive joint structural damage can still be observed in patients fulfilling clinical remission criteria^4^. Brown *et al*. carried out the first study to demonstrate the dissociation between clinical remission and radiographic progression in RA patients, and demonstrate a direct association between synovitis, as detected by musculoskeletal ultrasound (MSKUS) and radiographic progression in individual joints^5^. A large body of evidence suggests that the persistence of subclinical synovitis detected by MSKUS is associated with a high risk of radiological progression^6–8^. Regarding the persistence of subclinical synovitis, the concept of ultrasound remission has been proposed in addition to clinical remission^9,10^.

MSKUS is an ideal modality to detect early joint synovitis sensitively. The recommendations of European League Against Rheumatism (EULAR) suggest that ultrasound can detect inflammation, which predicts subsequent joint damage, even when clinical remission is present and can be used to assess persistent inflammation^11^. Animal models of autoimmune arthritis have indicated as valuable research tools for identifying potential pathogenic mechanisms and evaluating potential therapies for RA. Type II collagen-induced arthritis (CIA) is the most widely studied model of RA and shares several pathological features with RA^12^. Ultrasonography could even more accurately detect arthritis lesions in CIA mice^13^.

A recent study found that achieving ultrasound remission at 6 months was associated with no radiographic progression during the subsequent year^14^. Furthermore, in our primary experience, ultrasound remission soon after treatment is a predictive factor for achieving good therapeutic outcomes [these primary data were from an ongoing clinical trial (No: ChiCTR1900021850) and have not been published]. However, no study has explored ultrasound remission at which time point could predict good structural outcome. Therefore, the present study aims to determine whether ultrasound remission has predictive value and ultrasound remission at which time point has predictive value for good structural outcome.

## Results

### Clinical characteristics

**Figure 1:**
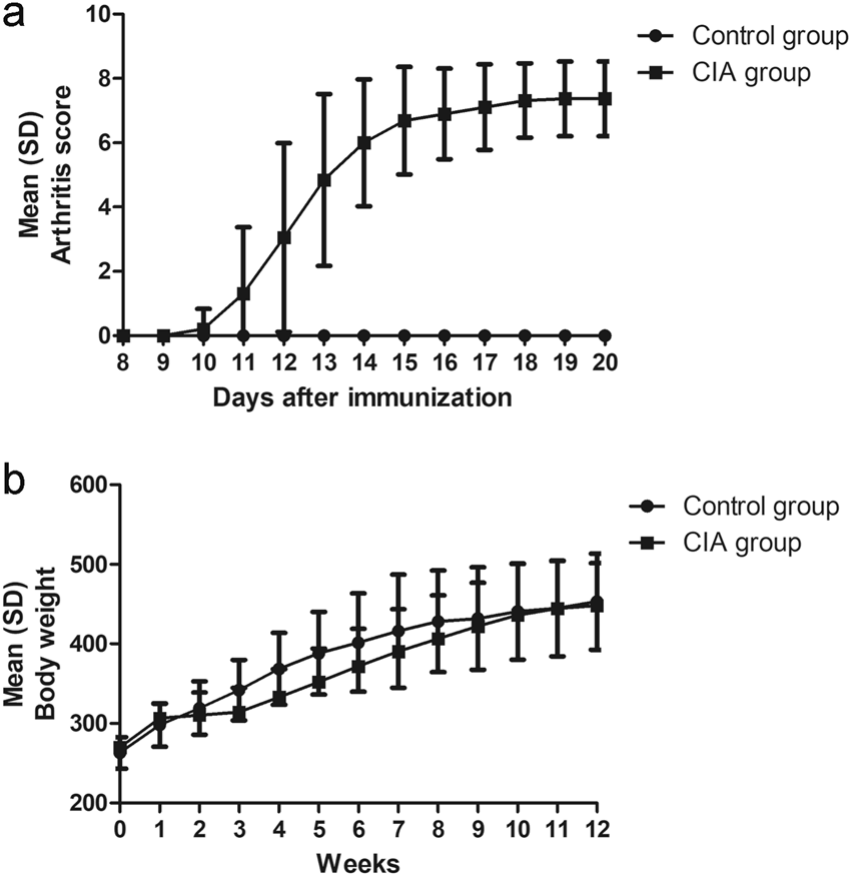
Changes in arthritic score (**a**) and body weight (**b**) after the first immunization in both the CIA group and the control group.

Beginning on day 11, the arthritis score for CIA rats increased progressively compared with the control group and reached a plateau on day 19. Arthritis developed in all rats at approximately day 15 after the first immunization. Beginning 3 weeks after the first immunization, the CIA rats gained significantly less weight than the control group (Fig. 1). There was no significant difference in body weight or arthritis score between the treated and untreated groups at baseline (see Supplementary Table S1).

### Ultrasound findings

Of the 48 joints in the treated CIA group, synovial hypertrophy was observed in 44 joints at baseline and in 29 joints at the end point, PD signals were observed in 25 joints at baseline and in 8 joints at the end point, and bone erosions were observed in 42 joints at baseline and in 27 joints at the end point. Of the 16 joints in the untreated CIA group, synovial hypertrophy was observed in 16 joints at baseline and in 16 joints at the end point, PD signals were observed in 6 joints at baseline and in 9 joints at the end point, and bone erosions were observed in 16 joints at baseline and in 16 joints at the end point (Table 1). In the treated group, the scores for synovial hypertrophy, PD signal and bone erosion decreased from baseline to the end. In the untreated group, there were no significant differences in synovial hypertrophy, PD signal or bone erosion scores from baseline to the end point (details can be found as a Supplementary Note online). At baseline, no significant differences were observed for synovial hypertrophy, PD signals or bone erosions between the two groups (P = 0.564, 0.392, and 0.323, respectively). At the end point, significant differences were observed for synovial hypertrophy, PD signals or bone erosions between the two groups (P = 0.002, 0.007, and 0.001, respectively).

**Table 1:** Joints with synovial hypertrophy, PD signals and bone erosions in CIA rats.

Groups	Grade	Joints with synovial hypertrophy, n/N(%)		Joints with PD signals, n/N(%)		Joints with bone erosions, n/N(%)	
		At baseline	At the end	At baseline	At the end	At baseline	At the end
Treated CIA rats	grade 0	4/48 (8.3)	19/48 (39.6)	23/48 (47.9)	40/48 (83.3)	6/48 (12.5)	21/48 (43.8)
	grade 1	6/48 (12.5)	17/48 (35.4)	18/48 (37.5)	7/48 (14.6)	16/48 (33.3)	22/48 (45.8)
	grade 2	17/48 (35.4)	10/48 (20.8)	5/48 (10.4)	1/48 (2.1)	12/48 (25.0)	3/48 (6.3)
	grade 3	21/48 (43.8)	2/48 (4.2)	2/48 (4.2)	0 (0)	14/48 (29.2)	2/48 (4.2)
Untreated CIA rats	grade 0	0 (0)	0 (0)	10/16 (62.5)	7/16 (43.8)	0 (0)	0 (0)
	grade 1	2/16 (12.5)	1/16 (6.3)	4/16 (25.0)	6/16 (37.5)	4/16 (25.0)	3/16 (18.8)
	grade 2	5/16 (31.3)	4/16 (25.0)	1/16 (6.3)	2/16 (12.5)	6/16 (37.5)	6/16 (37.5)
	grade 3	9/16 (56.3)	11/16 (68.8)	1/16 (6.3)	1/16 (6.3)	6/16 (37.5)	7/16 (43.8)

### Inter-observer agreement

Forty-eight ankle joints were examined by two investigators. Analyses of inter-observer agreement were shown in Table 2. The ICC and unweighted kappa estimations for the examined parameters showed a good correlation (0.73–0.91 and 0.63–0.75, respectively) between the two US investigators. The overall agreement was high (73–85%).

**Table 2:** Inter-observer agreement.

Ultrasonographic factors	ICC	Kappa	Overall agreement
Synovial hypertrophy	0.91	0.75	83.3
PD signal	0.73	0.71	85.4
Bone erosions	0.90	0.63	72.9

### Ultrasonographic factors at baseline associated with good structural outcome

At the joint level, possible factors predicting good structural outcome were synovitis for grey scale, PD signal, and bone erosion at baseline. Additionally, we defined good structural outcome as bone erosion grade ≤1 at 12 weeks after treatment. However, no ultrasonographic factors at baseline were univariately and multivariately significantly associated with good structural outcome at the joint level (Table 3).

**Table 3:** Univariate and multivariate analysis of the ultrasonographic factors at baseline associated with good structural outcome.

Ultrasonographic factors at baseline	Univariate		Multivariate	
	OR (95% CI)	P Value	OR (95% CI)	P Value
Synovial hypertrophy	6.12 (0.63 to 59.5)	0.119	4.43 (0.39 to 49.9)	0.229
PD signal	4.19 (0.43 to 40.6)	0.216	2.34 (0.20 to 26.9)	0.497
Bone erosions	3.82 (0.39 to 37.0)	0.248		

### Remission and good structural outcome

**Figure 2:**
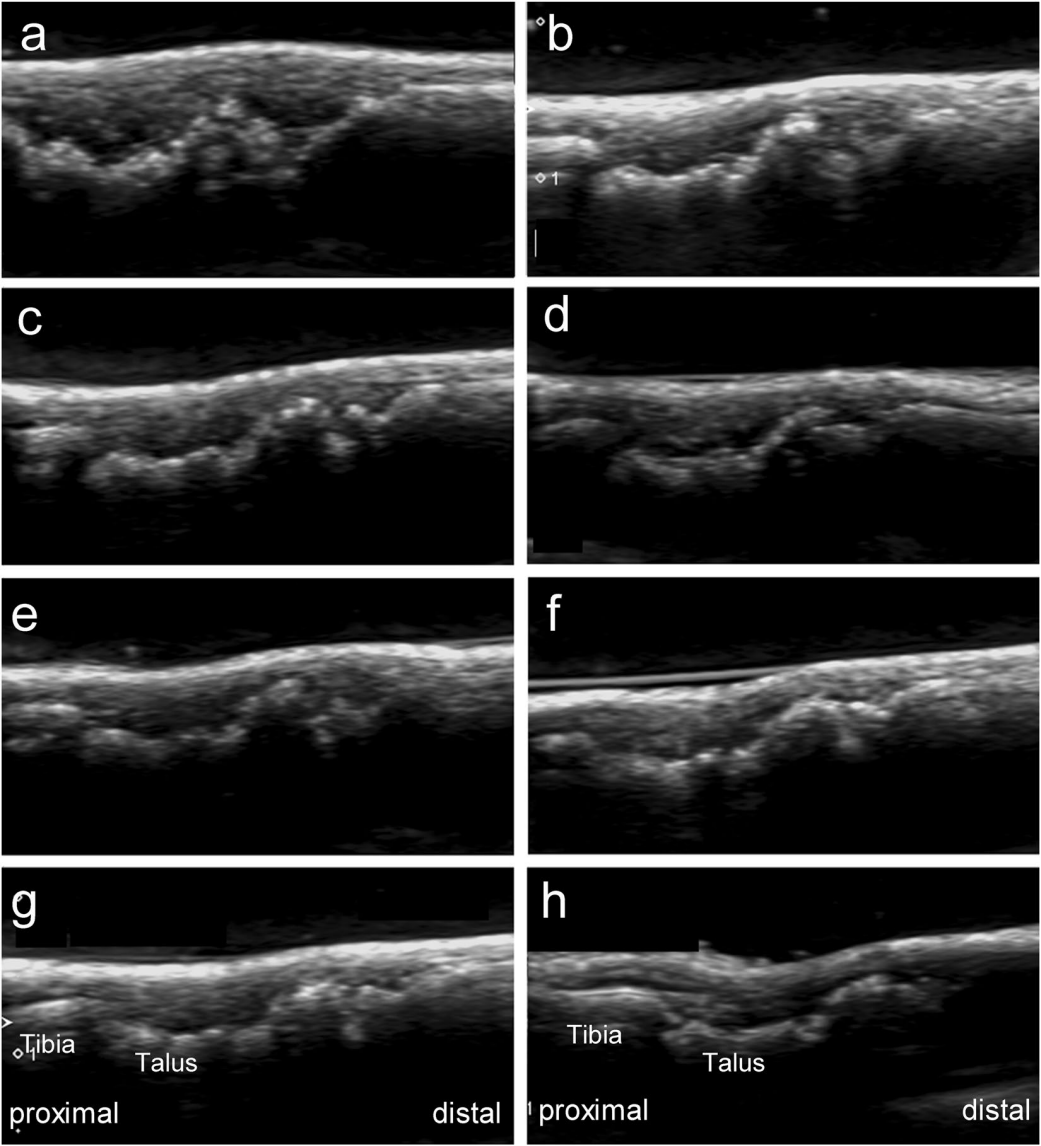
The left ankle of a CIA rat on ultrasonography at different points. (**a**) At baseline. (**b**) The first week after treatment. (**c**) The second week after treatment. (**d**) The third week after treatment. (**e**) The fourth week after treatment. (**f**) The fifth week after treatment. (**g**) The sixth week after treatment. (**h**) The twelfth week after treatment.

Among the 44 joints with synovitis of treated CIA rats, ultrasound remission was significantly associated with good structural outcome from 4 to 6 weeks after treatment. Ultrasound remission from 4 to 6 weeks after treatment had the highest area under the curve, sensitivity, specificity, and positive and negative predictive values to predict good structural outcome (Table 4, Fig. 2).

**Table 4:** The performance of ultrasound remission at different time points after treatment for identifying joints with good structural outcome.

Ultrasound remission at different time points	Prevalence of good structural outcome							
	Joints in remission n/N (%)	Joints not in remission n/N (%)	P Value	Sensitivity	Specificity	PPV	NPV	AUC
At 1 week after treatment	16/17 (94.1)	7/27 (25.9)	0.634	0.41	0.80	0.94	0.15	0.61
At 2 weeks after treatment	26/27 (96.3)	13/17 (76.5)	0.065	0.67	0.80	0.96	0.24	0.73
At 3 weeks after treatment	27/28 (96.4)	12/16 (75.0)	0.051	0.69	0.80	0.96	0.25	0.75
At 4 weeks after treatment	32/33 (97.0)	7/11 (63.6)	0.010	0.82	0.80	0.97	0.36	0.81
At 5 weeks after treatment	32/33 (97.0)	7/11 (63.6)	0.010	0.82	0.80	0.97	0.36	0.81
At 6 weeks after treatment	32/33 (97.0)	7/11 (63.6)	0.010	0.82	0.80	0.97	0.36	0.81

PPV, positive predictive value; NPV, negative predictive value; AUC, area under the curve.

## Discussion

We found that ultrasound remission from 4 to 6 weeks after treatment has a high value to predict good structural outcome in CIA rats. To our knowledge, this study is the first to assess the relationship between ultrasound remission at different time points and good structural outcome.

Our study found that ultrasonographic factors at baseline could not predict future structural progression in CIA rats. This finding is in line with a multicentre cohort study of newly diagnosed RA patients. Ten Cate *et al*. found that adding baseline ultrasonography did not improve the prediction of radiographical progression at 12 months in RA patients^15^. However, previous findings on the ability of baseline synovitis and bone erosion to predict structural damage were inconsistent. Dougados *et al*. observed that baseline synovitis increased the risk of structural progression in RA patients^16^. Funck-Brentano *et al*. found that baseline US erosions in RA patients were predictive of the presence of radiographic erosions at 1 year in a multicentre cohort study of early arthritis patients^17^. Moreover, Courbon *et al*. investigated the association of early bone loss with late arthritis severity in adjuvant-induced arthritis (AIA) rats and found that bone alterations on day 10 were strongly correlated with arthritis severity or bone loss on day 17^18^. However, the AIA rats in this study were not treated, which is different from our study. Therefore, the negative results of our study might be explained by early observation and intervention in the therapeutic window of opportunity. Recent studies have suggested there is a therapeutic window of opportunity in early RA, during which early treatment could alter the disease course and improve long-term outcomes^19,20^. Early therapeutic intervention (within 3 months of the onset of symptoms) in RA patients resulted in higher remission rates, improved clinical outcomes and reduced bone damage and joint disabilities compared with the results in patients who received delayed treatment^21,22^.

Notwithstanding that ultrasonographic factors at baseline could not predict future structural progression in CIA rats, baseline ultrasound may have clinical value and ultrasound remission after treatment may have predictive value for future progressive structural damage. A comparative study between clinical examination and ultrasound suggested that persistent synovitis after 4 months of therapy was predictive of subsequent structural progression^16^, but Dougados *et al*. did not provide a rationale for choosing the time point of 4 months and it is not clear whether the results would be plausible when another time point was chosen. Moreover, a recent study suggested that achieving ultrasound remission at 6 months was associated with no radiographic progression during the subsequent year, as well as ACR/EULAR Boolean remission^14^. However, this study only explored the predictive value of ultrasound remission at 6 months. Hence, whether ultrasound remission at different time points was associated with no radiographic progression remains unknown.

The strength of our study was to explore whether ultrasound remission at some specific time point has predictive value for good structural outcome. We found that ultrasound remission from 4 to 6 weeks after treatment has a high value to predict good structural outcome in CIA rats. We could assume that if ultrasound remission is achieved within some specific time period from the start of treatment in RA patients, attaining a good structural outcome at a subsequent follow up period is highly likely. However, there are some notable differences between CIA rats and RA patients, therefore, further research in RA patients is needed to explore the problem.

However, two recent multicentre randomized controlled clinical trials (ARCTIC and TaSER) have shown that a treatment strategy targeting ultrasound remission in early RA was not associated with significantly better clinical or imaging outcomes^23,24^. In other words, targeting ultrasound remission is not superior to targeting clinical remission or low disease activity in predicting the outcome of RA patients. Therefore, Caporali *et al*. proposed the idea of forgetting ultrasound and focusing on clinical assessment in RA management^25^. However, D’Agostino considered whether these studies are sufficient to definitively inform our practice in the current clinical context. Due to the different study designs and end points in recent studies, D’Agostino concluded that a robust evaluation of the usefulness of US in RA clinical practice is still needed^26^.

There are several limitations in the present study. First, due to the inconsistency of the starting treatment time and more uncertain factors in RA patients, we controlled the uniform starting treatment time in CIA rats in this study. Therefore, we could not compare the predictive role of ultrasound remission at different starting treatment times. However, we will divide CIA rats into several subgroups according to the beginning treatment time in future research. Second, given the preliminary consensus on the recognition of sonographic manifestations including synovial hyperplasia, PD signal and structure destruction, we examined the histological features of some of the rats. Therefore, our successive plan is to obtain pathological data for all CIA rats and use the histology as an outcome. Third, although we found ultrasound remission has a high value for predicting good structural outcome in CIA rats, some notable differences exist between this model and RA patients. Therefore, further research is needed to explore the value and potential mechanisms of ultrasound remission in RA patients. Fourth, CIA rats were not continually treated after the 6 weeks of treatment; therefore, for rats who remained unresponsive after 6 weeks, we did not compare structural outcomes between rats with continuation and discontinuation of therapy. Further therapeutic options remain important for patients who remain unresponsive to achieve the cessation of structure progression and disability.

In conclusion, ultrasound remission from 4 to 6 weeks after treatment has a high value to predict good structural outcome in CIA rats. However, there are still several problems with the implementation of ultrasound remission as a recommendation. Therefore, further research is needed to explore the predictive value of ultrasound remission in RA patients.

## Materials and Methods

### Experimental animals

Animal procedures were approved by the ethics committee of Peking University People’ s Hospital (protocol no. 2015-38), and the experiments were performed according to the ARRIVE (Animal Research: Reporting of *In Vivo* Experiments) guidelines and checklist^27^. Forty male Wistar rats, aged 5 to 6 weeks with a body weight of approximately 200 to 250 g, were purchased from Vital River Laboratory Animal Co. Ltd, Beijing, China. The rats were housed at two animals per cage in the Laboratory Animal Unit of Peking University People’s Hospital and acclimatized for 1 week under constant environmental conditions with 12 hour light/dark cycles.

### Induction of CIA

Bovine type II collagen (CII, 2 mg/ml, solution in 0.05 M acetic acid, Chondrex, Redmond, WA, USA) was diluted to 1 mg/ml with an equal volume of complete Freund’s adjuvant (CFA, Sigma-Aldrich, Taufkirchen, Germany) or incomplete Freund’s adjuvant (IFA, Sigma-Aldrich). Thirty-two rats were anaesthetized in air with isoflurane and immunized with 0.2 ml of bovine type II collagen-CFA emulsion subcutaneously at the base of the tail. On the 7th day of the study, a booster immunization with 0.1 ml of bovine type II collagen-IFA emulsion was administered at the base of the tail, but the injection site was proximal to the primary injection site. Eight rats were unimmunized and served as controls.

### Clinical assessment

Each hind paw was recorded on a scale of 0–4 as follows: 0 = no evidence of erythema and swelling 1 = erythema and mild swelling confined to the tarsals or ankle joint, 2 = erythema and mild swelling extending from the ankle to the tarsals, 3 = erythema and moderate swelling extending from the ankle to metatarsal joints, and 4 = erythema and severe swelling encompassing the ankle, foot and digits, or ankylosis of the limb. The maximum arthritic score per rat was set at 8.

### Experimental design

**Figure 3:**
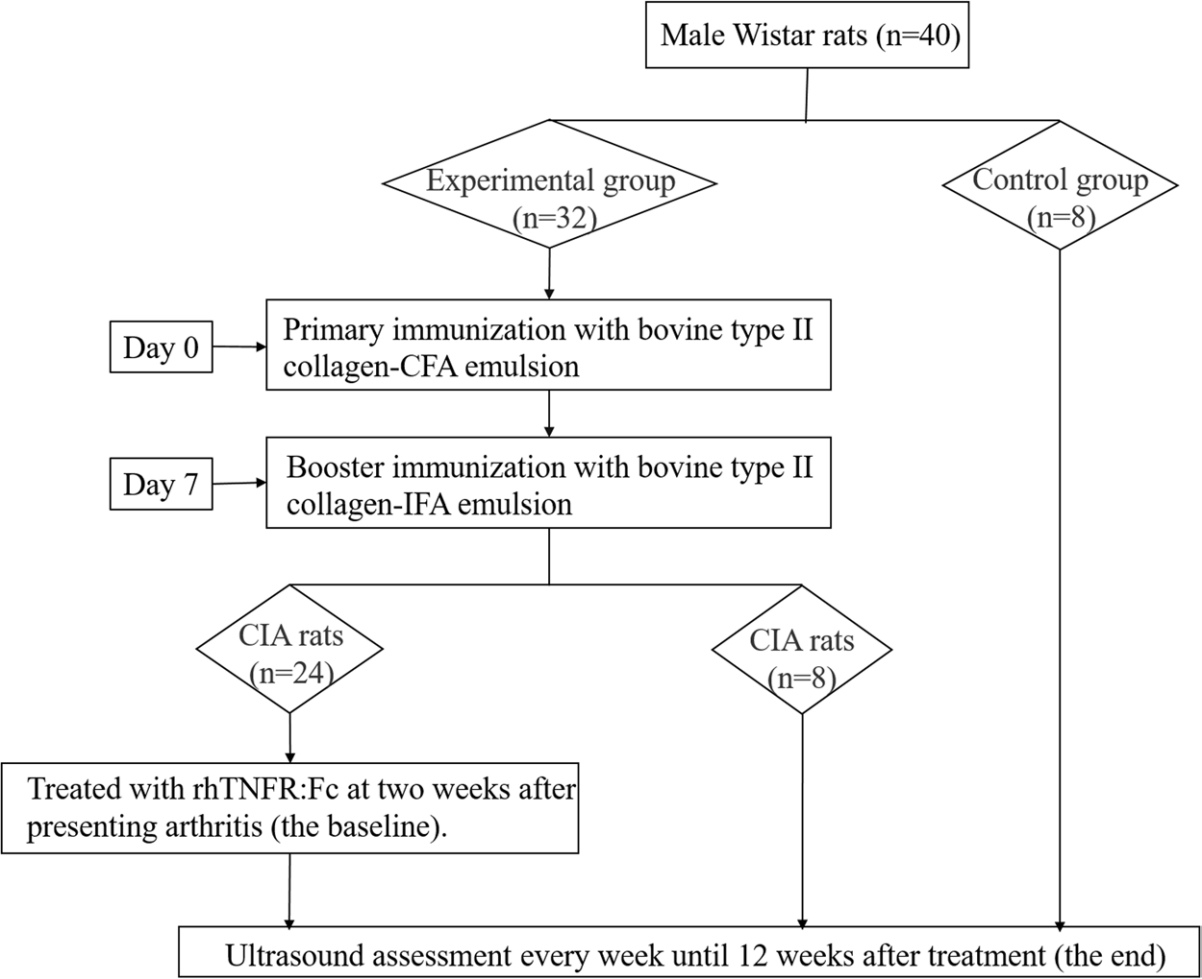
Flow chart of the experimental design.

Twenty-four of the 32 immunized rats were treated with rhTNFR:Fc (Shanghai CP Guojian Pharmaceutical Co., Ltd., Shanghai, China; 5 mg/kg, i.p., two times a week, for 6 weeks) at two weeks after presenting arthritis. The time point at which the CIA rats were treated was the baseline. Eight of the 32 immunized rats were left untreated. The baseline was also two weeks after presenting arthritis for untreated CIA rats. The rats were observed every day following the booster immunization until day 21, and then observed twice a week for clinical scores. Ultrasonography was performed every week following the booster immunization until 12 weeks after treatment (the end). The flow chart of the experimental design is shown in Fig. 3.

### Ultrasound assessment

Each rat was anaesthetized intraperitoneally (i.p.) with 50 mg/kg tiletamine/zolazepam (Zoletil 50, Virbac Lab, Carros, France) before ultrasound examination. Hairs were removed from the ankles using depilatory cream. The ankle joints of both hind paws of each rat were evaluated using an Aplio 500 TUS-A500 (Toshiba Medical Systems Corporation, Tochigi, Japan) with an 18 MHz broad band linear array transducer. The colour gain was set just below the level at which colour noise appeared on the underlying bone. All joints were scanned on the dorsal aspect. Ultrasonography was performed by an investigator with 5 years of experience in MSKUS. To study inter-observer agreement, another investigator with 3 years of experience in MSKUS and blinded to the results of previous measurements, performed the ultrasound examinations in 24 CIA rats at baseline. Prior to the study, the investigators reached consensus with regard to the scoring system. The investigators were not aware of the clinical characteristics and previous ultrasound results. The ultrasound assessment consisted of synovial hypertrophy, PD signal, and bone erosion using a semiquantitative scale (0–3)^28^. The details for the scoring system and ultrasonographic appearances (synovial hypertrophy, PD signal and erosions) of the ankles of CIA rats can be found as Supplementary Figs S1–S3. We defined ultrasound remission as grey scale grade ≤1 without PD signal.

### Statistical analysis

Clinical and ultrasound data were presented as proportions (%). The differences in treatment effects between CIA groups were compared by using the chi-square test. Inter-observer agreement was calculated by intraclass correlation coefficients (ICC), unweighted kappa statistics, and overall agreement. A kappa value of 0–0.20 was considered poor; 0.21–0.40, fair; 0.41–0.60, moderate; 0.61–0.80, good; and 0.81–1.00, excellent agreement. Univariate and multivariate regression analyses were used to analyse possible variables of good structural outcome. Factors that have been shown to have prognostic value in the previous literature, were included in the multivariate analyses. A Chi-square test was performed to explore the predictive values of ultrasound remission at different time points for good structural outcome. Finally, we calculated the area under the curve, sensitivity, specificity, and positive and negative predictive values of ultrasound remission at different time points. All statistics were performed by using SPSS 18.0 software. A value of P < 0.05 was considered statistically significant.

### Ethical approval and informed consent

Animal procedures were approved by the ethics committee of the Peking University People’s Hospital, and the experiments were performed according to the ARRIVE (Animal Research: Reporting of *In Vivo* Experiments) guidelines and checklist.

## Supplementary information

Supplementary information

## Data Availability

The datasets generated and/or analysed during the current study are available from the corresponding author on reasonable request.
