# Supplementary material for: Ultrasound remission can predict future good structural outcome in collagen-induced arthritis rats
Source: Sci Rep. 2019 Sep 16;9:13294. doi: 10.1038/s41598-019-49948-7 (PMC6746853; doi:10.1038/s41598-019-49948-7)
Supplement: Supplementary file 1 — Supplementary information [file 41598_2019_49948_MOESM1_ESM.pdf]

Ultrasound remission can predict future good structure outcome in collagen-induced arthritis rats

Wenxue Li+, Yiqun Liu+, Jiaan Zhu*, Arong Bilig, Fang Liu, Zheng Chen.
Department of Ultrasound, Peking University People' s Hospital, Beijing, China.
* Corresponding. author (email: zhujiaan@pkuph.edu.cn)
+ These authors contributed equally to this work.

Results

Clinical characteristics (Supplementary Table S1)

Basic characteristics at baseline	Treated CIA rats	Untreated CIA rats	P Value
Body weight	310.63 ± 28.09	297.50 ± 33.23	0.547
Arthritis score	7.29 ± 1.21	7.50 ± 0.71	0.820

Supplementary table S1. Basic characteristics at baseline of the treated and untreated CIA rats

Ultrasound findings. (Supplementary Note)

In the treated group, the scores for synovial hypertrophy, PD signal and bone erosions decreased from baseline to the end point. Six (12.5%) scores for synovial hypertrophy decreased from Grade 1 to 0, 6 (12.5%) decreased from Grade 2 to 0, 9 (18.8%) decreased from Grade 2 to 1, 3 (6.3%) decreased from Grade 3 to 0, 8 (16.7%) decreased from Grade 3 to 1, and 8 (16.7%) decreased from Grade 3 to 2. Sixteen (33.3%) scores for PD signals decreased from Grade 1 to 0, 5 (10.4%) decreased from Grade 2 to 1, 1 (2.5%) decreased from Grade 3 to 2, and 1 (2.5%) decreased from Grade 3 to 0. Twelve (12.5%) scores for bone erosions decreased from Grade 1 to 0, 4 (12.5%) decreased from Grade 2 to 0, 6 (18.8%) decreased from Grade 2 to 1, 12 (6.3%) decreased from Grade 3 to 1, nevertheless, 1 (16.7%) increased from Grade 0 to 1, and 1 (16.7%) increased from Grade 1 to 2.

In the untreated group, there were no significant differences in synovial hypertrophy, PD signal or bone erosion scores from baseline to the end point. One (6.3%) score for synovial hypertrophy increased from Grade 1 to 2, and 2 (12.5%) decreased from Grade 2 to 3. Two (12.5%) score for PD signals decreased from Grade 1 to 0, 4 (25.0%) increased from Grade 0 to 1, and 1 (6.3%) increased from Grade 0 to 2. One (6.3%) score for bone erosions increased from Grade 1 to 2, and 1 (6.3%) increased from Grade 2 to 3.

Materials and Methods

Ultrasound assessment (Supplementary Figure S1-S3)

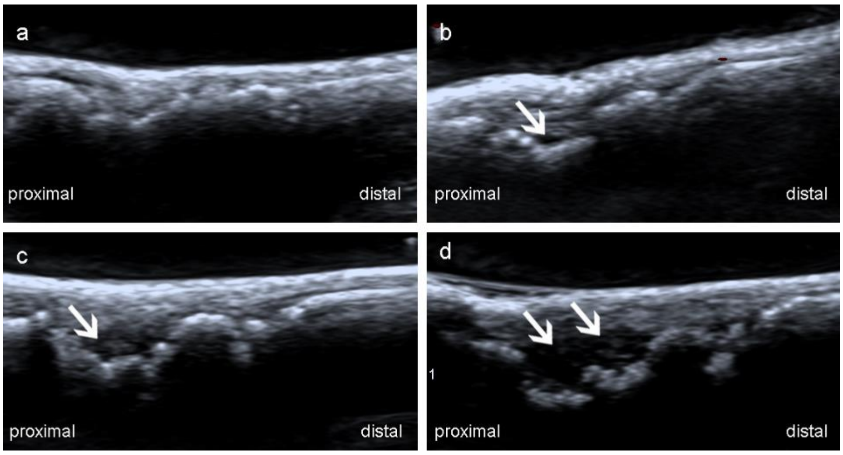

Supplementary Figure S1. Synovial hypertrophy of ankles in CIA rats on ultrasonography. a Grade

0, no synovial thickening; b grade 1, minimal synovial thickening (filling the angle between the periarticular bones, without bulging over the line linking tops of the bones [arrow]); c grade 2, synovial thickening bulging over the line linking tops of the periarticular bones but without extension along the bone diaphysis (arrow); d grade 3, synovial thickening bulging over the line linking tops of the periarticular bones and with extension to at least one of the bone diaphysis (arrow).

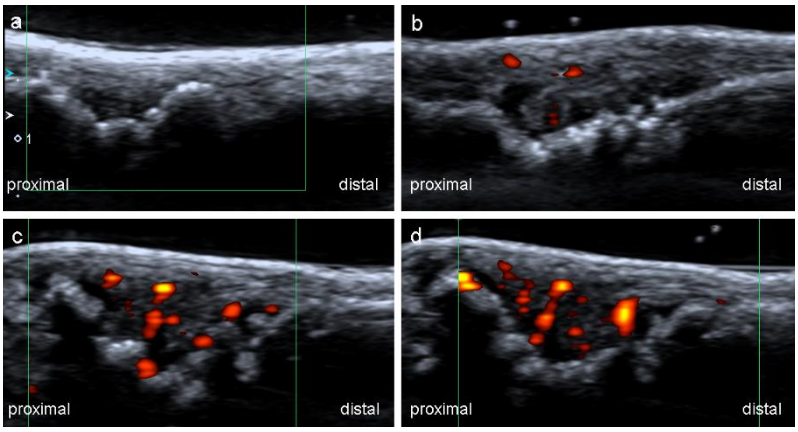

Supplementary Figure S2. PD signal of ankles in CIA rats on ultrasonography. a Grade 0, no flow in the synovium; b grade 1, single vessel signals; c grade 2, confluent vessel signals in less than half of the area of the synovium; d grade 3, vessel signals in more than half of the area of the synovium.

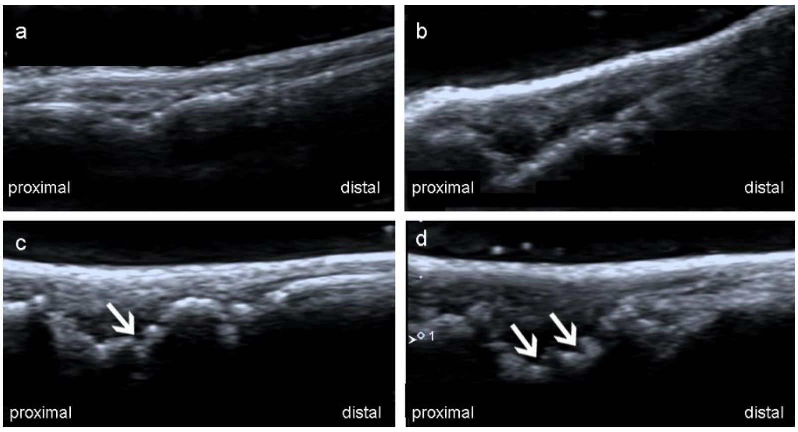

Supplementary Figure S3. Bone erosions of ankles in CIA rats on ultrasonography. a Grade 0, regular bone surface; b grade 1, irregularity of the bone surface without formation of a defect seen in 2 planes; c grade 2, formation of a defect in the surface of the bone seen in 2 planes (arrow); d grade 3, bone defect creating extensive bone destruction (arrows).
